# How family sports behavior shapes adolescent sleep: mediating effects in causation

**DOI:** 10.3389/fpubh.2024.1518960

**Published:** 2025-01-17

**Authors:** Wenqi Wu, Aoming Xie, Xiaotian Li, Yiran Xun

**Affiliations:** ^1^School Physical Education, Sports Sociology, Capital University of Physical Education and Sports, Beijing, China; ^2^Gymnastics, Sports Education and Training, Xian Physical Education University, Xian, China

**Keywords:** sleep, adolescents’ physical activity, family physical behavior, two-stage least squares, IV-based causal mediation effect

## Abstract

**Objective:**

This study aimed to describe adolescent sleep and explore its association with physical activity, focusing on the mediating role of family physical behavior.

**Methods:**

Data were sourced from the Chinese Adolescent Health Database, which included responses from 7,482 adolescents aged 12–18 years about sleep, personal physical activity, and family physical activity. Using two-stage least squares (2-SLS), we examined the relationship between adolescent sleep, personal physical activity, and father’s physical activity habits (as instrumental variables). A mediating variable, family physical behavior, was introduced to investigate causal pathways.

**Results:**

2-SLS regression revealed a significant positive correlation between physical activity and sleep quality (*β =* 0.052, *p*<*0.01*). With the inclusion of the instrumental variable (father’s physical activity habits), the effect size increased to 0.208 (*p*<*0.01*). Adding family physical behavior as a mediating variable further increased the coefficient by 0.1437. The analysis showed that 84.62% of the total effect of physical activity on sleep was mediated, while the direct effect accounted for only 15.53%. Both F-statistics were significant.

**Conclusion:**

Family physical behaviors significantly enhance the positive impact of physical activity on adolescent sleep. Promoting family sports participation is essential for fostering healthy lifestyles and improving adolescent sleep quality. This highlights the need for interventions that incorporate family physical activities into adolescent health strategies.

## Introduction

Sleep is a fundamental part of human life activities, and one-third of a person’s life is spent in sleep. Essentially, sleep is a restorative process that relieves the body of fatigue and restores the body’s spirit. Recently, the American Academy of Sleep Medicine (AASM) recommended that adolescents between the ages of 13 and 18 get 8 to 10 h of sleep per day to promote optimal physical and mental health (1). However, according to the World Health Organization (WHO) in recent years, the global rate of sleep disorders has reached 27%, and this rate continues to rise. In addition, more than 90 percent of teens do not meet the recommended standard for hours of sleep. A study published in 2022 noted that adolescents who slept less than 9 h per night had significantly smaller volumes in key areas of the brain responsible for memory, intelligence, and happiness, such as the hippocampus and prefrontal cortex, compared to adolescents who had a healthy amount of sleep per night (2). This suggests that chronic sleep deprivation can have an irreversible negative impact on the structure of a teenager’s brain, which can affect learning ability, emotional regulation, and overall mental health. According to a University of California study, teens who averaged less than 6 h of sleep had standardized test scores that were about 20 percent lower than their peers who slept the recommended amount of time (3). It has also been shown that sleep deprivation in adolescents is also associated with a significant increase in depressive symptoms, especially at the high school level, where the risk of depression is three times higher for sleep-deprived students than for those who get enough sleep (4). Adolescence is a critical period of physical and mental development for adolescents, and adequate sleep is especially important for adolescents (5, 6). Studies have shown that chronic sleep deprivation weakens immune system function and increases the risk of obesity, diabetes, and mental illnesses such as anxiety and depression. For example, a large epidemiologic study found that adolescents who slept less than 7 h per night had a body mass index (BMI) that was on average 10% higher than adolescents who had adequate sleep, with a significant increase in the risk of obesity (7). In addition, a team of researchers at the University of Michigan noted that sleep deprivation can lead to a decrease in the activity of immune cells in adolescents, making them more susceptible to common illnesses such as the flu (8).

Just as health is a multidimensional concept, so is sleep. Carskadon and Dement define sleep as a recurrent, reversible neurobehavioral state accompanied by a disengagement of relative perception of the environment, including human actions such as postural recumbency, behavioral quiescence, and eye closure (9). However, the sleep studied in this paper is more oriented to sleep health, which is a multidimensional “sleep-wakefulness-fullness” model. It has been found that in adolescent populations, good sleep health patterns (subjective satisfaction, appropriate timing, adequate duration, high efficiency, and sustained alertness during waking hours) may be adversely affected, especially adequate duration (10). For example, adolescent electronic device use (11), parent-set bedtimes (12), and school start times (13) can affect the duration of adolescent sleep. Carskadon suggests that the intersection of later bedtimes and earlier school start times creates what is known as the “perfect storm” of teenage sleep (14). Physical activity may be associated with sleep. Studies have shown that there is a bidirectional correlation between physiology and sleep, and that physical activity can alleviate the problem of sleep disorders in people by regulating their physiology and mood (15). In particular, moderate physical activity has beneficial effects on sleep in adults (16). For example, daily walking exercise is beneficial in improving physical activity and subjective sleep quality in adults (17). A similar correlation exists between mental health and sleep, with physical activity being an effective means of relieving psychological pressures such as academic, peer, and social pressures in adolescents, and adolescents who participate in weekly physical activity have less psychological stress and a higher sense of well-being (18). At the same time, adolescents can also develop their strong will qualities and strong psychological endurance by experiencing emotions such as success and failure during physical exercise (19). In addition, the literature has explored the impact of factors such as family parenting behaviors (20) and family functioning on adolescent sleep. Studies have found that parental behavior, cognitive emotions, parent–child relationships and parental well-being and psychopathology, as well as parents’ socio-cultural backgrounds (20) affect adolescent sleep. Moreover, disruptive family routines that can disturb adolescents’ sleep activities, as well as parental monitoring of children’s behavior and enforcement of sleep-related rules are positively associated with adolescent sleep (21). Also participating in physical activity with parents significantly improves the quality of sleep in adolescents (22). Family physical activity behaviors not only enhance the emotional connection between family members, but also increase the encouragement and supervision of youth physical activity.

Based on the summary and combing of the established literature, the author found that most of the existing studies utilized the research method of causal inference to empirically measure the relationship between physical activity, family and sleep, and to accumulate valuable experience for improving sleep quality. However, there are still three points of excavation regarding the relationship between physical activity and sleep in adolescents: First, in terms of research subjects, the established literature focuses mainly on people with sleep disorders and adults, with slightly less attention paid to adolescents; Second, in terms of research topics, the established literature on adolescent physical activity and sleep focuses mainly on adolescent psychosocial and social pressures, and pays slightly less attention to improving adolescent sleep through family physical activity behaviors; Finally, in terms of research methodology, the established literature mainly uses linear regression models for causal inference, but the use of mediator and instrumental variable methods is slightly underutilized. Accordingly, this study investigates the potential associations between sleep and adolescent physical activity and fathers’ physical activity habits (instrumental variables) through two-stage least squares (2-SLS) by combing the existing studies, and adds a mediator variable (family physical activity behaviors) on this basis to investigate the causal paths between sleep and physical activity, which bridges the gaps in the existing literature in terms of the subject matter, themes, and methods of the study. Therefore, the objectives of this study were (1) to explore the relationship between adolescent sleep and physical activity and (2) to assess the effects of fathers’ exercise habits, and family sports behaviors on adolescent sleep.

## Methods

### Data sources

The data in this article were obtained from the China Adolescent Health Database (23). The database uses field tests, questionnaires, and data collection to obtain data on adolescent health resources, including multi-wave surveys for the 2015/2016, 2016/2017, 2017/2018, and 2020/2021 school years. The purpose of this survey was to understand the current health status and health-related behaviors of middle and high school students in China. This dataset represents the first publicly shared dataset on adolescent health and health-related behaviors in China.

For policymakers, educational institutions, and other stakeholders, developing or adapting existing strategies to improve the well-being of China’s youth can be valuable and beneficial. The data include youth fitness test data and body composition data from 2015 to 2020 in 17 cities in Shandong Province. Anomalous data were excluded according to the following principles: (1) Anomalous data that deviates significantly from the actual situation due to the measurement and entry process; (2) Missing data in the database. A total of 9,048 samples of valid data were collected, 1,546 samples with missing variables were deleted, and the final number of samples retained was 7,482. The samples were between 12 and 18 years old, covering areas of different levels of economic development in Shandong, and were highly representative. The students surveyed gave informed consent and complied with ethical requirements.

### Selection and definition of variables

The dependent variable in this paper is sleep, which was measured based on the questionnaire “How many hours of sleep do you usually get each night?” to measure the subjects’ sleep. The pattern of good sleep health includes five aspects: subjective satisfaction, appropriate timing, adequate duration, high efficiency, and sustained alertness during waking hours (10). However, in this paper, the sleep focus is on sleep duration. Based on the National Sleep Foundation’s definition of adolescents, we define sleep deprivation as <8 h of sleep per night (24).

The independent variable in this paper is physical activity, which is measured by the questionnaire “On how many days in the past 7 days have you exercised for more than 30 min? (including all types of exercise that increase your heart rate and make you short of breath) to measure the physical activity of the subjects and assign a value of 1–8 to each option. In accordance with the World Health Organization guidelines on physical activity for adolescents (25), this paper defines a good physical activity profile as the number of days of physical activity per week ࣙ ≥3 for an individual adolescent.

The instrumental variable in this paper is the father’s physical activity habits (26–28), based on the questionnaire “How many times a week does your father exercise?” to measure fathers’ physical activity and assign a value of 1–5 to each option. In accordance with the World Health Organization guidelines on physical activity in adults (25), this paper defines a good physical activity profile as the number of days of physical activity per week ࣙ ≥ 2 for an adult individual.

The mediating variable in this paper is family physical behavior (29, 30), based on the questionnaire “How many times a week do you and your parents exercise together?” to measure the sporting climate in each family and assign a value of 1–5 to each option. Based on the physical activity requirements of adolescents and adults, this paper synthesizes the definition of a household with ≥2 days of physical activity per week as a good physical activity situation.

Based on a review of the established literature (31, 32), this paper used adolescents’ gender, year of birth, self-reported health status, and grade level as control variables. Among other things, the health status self-survey controls for the effects of other diseases on sleep in adolescents.

In terms of specific analyses, the average sleep duration of adolescents is 7.6 h, which indicates that there is a general lack of sleep among adolescents, especially among students in the junior and senior high school age groups, who generally do not achieve the desired sleep duration. In addition, according to the World Health Organization’s recommendations, children and adolescents should be physically active for an average of at least 60 min per day with moderate to vigorous intensity during the week. However, the data show that adolescents exercise an average of 3 days per week, which suggests that there is a general lack of exercise in the adolescent population and that overall physical activity among adolescents is unsatisfactory. Please refer to Table 1.

**Table 1:** Table of descriptive statistical analysis of the sample.

Variant	Definitions and assignments	Range of values	Mean	SD
Dependent Variable				
Sleep	How many hours of sleep do you usually get each night?(24-h day)		7.571	1.34
Independent Variable				
Physical exercise	How many of the last 7 days did you work out for more than 30 min?(Include all types of exercise that increase your heart rate and make you short of breath)	1–8	3.264	2.182
Instrumental Variable				
Physical habits of fathers	How many times a week does your dad exercise?	1–5	3.297	1.492
Mediary Variable				
Family physical Behavior	How many times a week do you and your parents exercise together?	1–5	2.49	1.379
Control Variable				
Gender	Male = 1, Female = 2	1–2	1.491	0.5
Birth year	Controlling for age of respondents based on year of birth	1991–2013	2005	1.884
Self-examination of health status	The following questions relate to your overall health. Overall, how do you think your health is?(from lowest to highest: excellent; very good; good; fair; poor)	1–5	2.287	1.028
Grade	Middle school = 1, high school = 2	1–2	1.382	0.486

### Modeling

#### Two-stage least squares

The two-stage least squares (2-SLS) method is a common method of causal inference that is particularly applicable in models with endogeneity problems. In this study, adolescents’ physical activity may be influenced by other unobserved variables (e.g., level of family education, individual health status, etc.), and these unobserved variables may also directly affect sleep, thus presenting the problem of omitted variables and making the Ordinary Least Squares (OLS) estimation results unreliable. Therefore, instrumental variables were introduced in this study to effectively address the endogeneity problem caused by reverse causality or omitted variables and to more accurately assess the causal relationship between physical activity and sleep. The applicability of the selection of fathers’ physical activity habits as an instrumental variable lies: (1) The instrumental variable (physical activity habits of fathers) was highly correlated with the endogenous variable (physical activity of adolescents), satisfying the correlation condition for the instrumental variable. Social learning theory suggests that parents’ behavioral habits have a significant role modeling effect on adolescents. Fathers’ frequent participation in physical activity may motivate adolescents to form similar exercise habits, establishing a high correlation between the two; The Behavioral Influence Model suggests that patterns of healthy behaviors in the family environment (e.g., fathers’ physical activity) significantly influence adolescents’ behavioral choices, particularly in the area of physical activity. (2) The instrumental variable does not directly affect the dependent variable (sleep), i.e., the effect of fathers’ physical activity habits on adolescents’ sleep must be realized indirectly through physical activity, fulfilling the assumption of exogeneity of the instrumental variable. A review of the literature did not identify studies in which fathers’ physical activity habits directly affected adolescent sleep. Thus, the main path of influence of this variable is indirectly affecting sleep through adolescent physical activity, which fulfills the condition of exogeneity of the instrumental variable. The specific model is constructed as follows:

(1) $$X=\alpha_{0}+\alpha_{1}Z+\in_{1}$$

(2) $$Y=\beta_{0}+\beta_{1}\hat{X}+\in_{2}$$

Among, X represents the independent variable physical activity, Y represents the dependent variable sleep, Z represents the instrumental variable father’s physical activity habits, and $\hat{X}$ is the first stage estimate of X . In addition, the model controls for other variables such as the adolescent’s gender, birth year, Self-examination of health status, and grade level.

#### The IV-based causal mediated effects model

The two-stage least squares method can only analyze the causal effect of adolescent physical activity and sleep, and the influence of father’s physical activity habits on this, for both causal effects of adolescent physical activity, family physical behavior and sleep of the role of the pathway cannot be identified synchronously, based on this, the introduction of IV of the causal mediating effect models. In the traditional framework of mediated effects analysis, it is usually assumed that the Treatment variable T and the Mediator variable M are exogenous, i.e., they are not influenced by other factors. However, if the treatment variable T and the mediator variable M are endogenous, i.e., they are influenced by other intrinsic factors, then mediation effects analysis requires special treatment. The IV-based mediated effects model serves to address the endogeneity of the treatment and mediating variables by using one instrumental variable for both causal and mediated effects analysis, ensuring more reliable and accurate results in mediated effects analysis, and thus a better understanding of the causal relationships between variables. In order to further analyze the transmissible role of family physical activity behavior in individual physical activity and sleep, an IV-based causal mediated effects model was used in this paper.

This paper uses family physical activity behavior as a mediating variable because after adolescent physical activity controls endogenous problems through the father’s physical activity habits, it also affects sleep through the mother’s physical activity habits, the family sports climate, and family attitudes toward sports. Based on this, this study constructed a linear probability model with sleep as the dependent variable, physical activity as the independent variable, and father’s physical activity habits as the instrumental variable as follows:

(3) $$X=\pi_{0}+\pi_{1}Z+\in_{1}$$

(4) $$M=\delta_{0}+\delta_{1}\hat{X}+\in_{2}$$

(5) $$Y=\beta_{0}+\beta_{1}\hat{X}+\beta_{2}\hat{M}+\in_{3}$$

Among, X represents the independent variable physical activity, Y represents the dependent variable sleep, Z represents the instrumental variable father’s physical activity habits, M represents the mediator variable family physical behavior, $\hat{X}$ and $\hat{M}$ are the estimates of the first and second stage, $\pi_{0}$ , $\pi_{1}$ , $\delta_{0}$ , $\delta_{1}$ , $\beta_{0}$ , $\beta_{1}$ , $\beta_{2}$ are the model parameters, and $\epsilon_{1}$ , $\epsilon_{2}$ , $\epsilon_{3}$ are the error terms.

## Results

### Two-stage least squares regression

Model 1 constructed a baseline regression model for sleep and physical activity to test the relationship between sleep and physical activity. The results showed a significant positive correlation between physical activity and sleep quality with a coefficient of 0.052 (*β =* 0.052, p<*0.01*), indicating that physical activity improves the sleep quality of individuals, and that for every unit increase in physical activity, the quality of sleep correspondingly increases by 0.052. See Table 2. To further explore the association between sleep and physical activity, a second-stage linear regression model2 with the addition of instrumental variables was constructed to test the relationship between sleep and physical activity. The results of the first stage regression showed that fathers’ physical activity habits had a significant positive effect on children’s physical activity behavior (*β =* 0.293, *p<0.01*). The second-stage regression showed that the coefficient of the effect of physical activity on sleep increased to 0.208 (*β =* 0.208, *p<0.01*), which was significantly higher than the results of the baseline model. The coefficient of the effect of physical activity on sleep increased from 0.052 to 0.208, indicating that the positive effect of physical activity on sleep was more pronounced after controlling for potential endogenous biases. This is because the choice of father’s physical activity habits as an instrumental variable more accurately reflects the independent effect of physical activity, thus revealing a strong causal relationship between physical activity and sleep. Also, the introduction of instrumental variables takes into account the influence of the father’s exercise habits on his children’s exercise behavior, directly avoiding the interference of other unobserved factors affecting sleep. Please refer to Table 2. Considering the effect of individual differences on the results, Model 3 incorporated four control variables, gender, year of birth, health self-assessment, and grade level, based on Model 2. Stage 1 results showed that females had significantly lower sleep quality than males (*β = −*0.337, *p<0.01*); the older the age, the worse the quality of sleep (*β = −*0.026, *p<0.01*); and adolescents with lower levels of fitness had poorer quality of sleep, a trend that was reflected in the grades of fitness level, step by step; adolescents in higher grades had significantly lower sleep quality (*β = −*0.123, *p<0.01*). The results of the second stage showed that the effect of physical activity on sleep quality remained significant and consistently increased even after accounting for individual differences (*β =* 0.209, *p<0.01*). This result reinforces the conclusion that physical activity has a positive effect on improving sleep quality and suggests that this positive effect remains stable even after accounting for individual differences. Please refer to Table 2.

**Table 2:** Table of results of two-stage least squares regression models.

Variant	Model 1	Model 2		Model 3	
	Two-stage sleep	Phase I	Phase II	Phase I	Phase II
Physical exercise	0.052^***^ (−0.004)		0.208*** (−0.022)		0.209^***^ (−0.021)
Fathers’ weekly exercise		0.293^***^ (−0.010)		0.284^***^ (−0.010)	
Gender				−0.337^***^*** (−0.03)	−0.049^***^ (−0.019)
Age				−0.026^***^ (−0.012)	0.160^***^ (−0.008)
Health Level II			−0.327^***^ (−0.041)		−0.049^*^
					(−0.025)
Health Level III				−0.560^***^ (−0.046)	−0.241^***^ (−0.031)
Health Level IV				−0.733^***^ (−0.052)	−0.468^***^ (−0.037)
Health Level V				−0.681^***^ (−0.129)	−1.092^***^ (−0.121)
Grade				−0.123^***^ (−0.045)	−0.463^***^ (−0.029)
Aggregate effect	7.402^***^ (−0.017)	2.291^***^ (−0.036)	6.893^***^ (−0.072)	54.454 (−23.441)	−314.074^***^ (−15.39)
*R2*	0.007	0.04		0.061	0.121
*N*	20,956			20,286	

*, ** and *** indicate significance at the 10, 5and 1% statistical levels, respectively; robust standard errors are in parentheses.

In summary, the effect of physical activity on sleep increased from 0.052 to 0.208 from Model 1 to Model 2, controlling for endogeneity bias, and was strongly significant. This suggests that the baseline model may underestimate the actual effects of physical activity. The coefficient of the effect of physical activity was further stabilized after adding control variables (Model3), indicating that controlling for potential confounding variables did not weaken the effect of physical activity on sleep, but rather verified its robustness. In addition, in terms of model fit, model 3 had the highest fit (*R* (2)*= 0.121*), indicating that the nested model with the inclusion of instrumental and control variables better explains the effects of physical activity on sleep and its results are highly reliable.

### IV-based modeling of causal mediating effects

**Figure 1:**
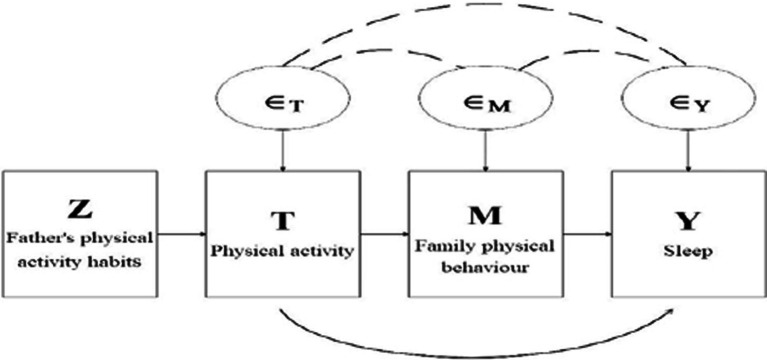
Diagram of IV-based model of causal mediating effects. Boxes indicate observable variables, circles indicate unobservable variables, arrows indicate causal relationships, and dotted lines indicate correlations.

Families play an important role in the development of adolescents, and parental support and encouragement are very important for adolescents’ physical activity and sleep. Therefore, based on the 2-SLS model, this paper chose family physical activity behavior as the mediating variable and father’s physical activity habit as the instrumental variable to analyze the mediating effect of sleep and physical activity, and the specific model is shown in Figure 1.

The results showed that the total effect value of physical activity on sleep was 0.2080 (*p<0.01*),which indicates that for every unit increase in physical activity among adolescents, the length of sleep increased by 20.80%. The direct effect value of 0.0323 (*p<0.01*), a relatively small but still significant coefficient, suggests that physical activity itself directly improves sleep quality. While the mediating effect value was as high as 0.1760 (*p<0.01*), we compared the direct effect value with the indirect effect value and found that the mediating effect of physical activity on sleep accounted for as high as 84.62% of the total effect, while the direct effect accounted for only 15.53% of the total effect, which further indicated that most of the effects of physical activity on sleep were realized through the mediating pathway. With the addition of family sports behavior, the coefficient of the effect of physical activity on sleep increased by 0.1437, which indicates that the effect of physical activity on sleep can be significantly enhanced through the intervention of family sports behavior, and that family sports behavior plays a key role in the improvement of physical activity on sleep. Joint physical activity among family members increases adolescents’ motivation to participate in exercise, while parents’ physical activity habits cause their children to indirectly improve sleep quality through behavioral imitation or environment creation. Please refer to Table 3.

**Table 3:** IV-based causal mediator analysis table.

Variant	Physical activity		
	Total effect	Direct effect	Indirect effect
Sleep	0.208^***^	0.0323^***^	0.176^***^
	(−9.570)	(−6.580)	(−7.400)
The first stage F1 statistic	881.212		
The second stage F2 statistic	4293.839		
*N*	20,956		

*, ** and *** indicate significance at the 10, 5and 1% statistical levels, respectively; robust standard errors are in parentheses.

In addition, on top of the traditional mediated effects analysis, the IV-based mediated effects model reports two F-statistics to assess the correlation of instrumental variables in the two-stage regression, respectively. The Stage 1\u00B0F-statistic was used to test the correlation between the instrumental variable and the treatment variable (core independent variable: youth physical activity), and weak instrumental variable identification was performed by reporting the corresponding Stage 1\u00B0F-statistic, with an *F*-value (*F>10*) indicating that the instrumental variable was a strong instrumental variable. The second stage F statistic was used to test the strength of explanation of the instrumental variables through the treatment variables for the mediating variables. The F-value (*F>30*) indicates that the instrumental variable is not only strongly correlated with the treatment variable but also has a significant effect on the mediator variable through the treatment variable. The F-statistic for the first stage was 881 (*F>10*) and the F-statistic for the second stage was 4,293 (*F>30*), which indicates that the father’s physical activity habits not only directly affect the adolescent’s individual physical activity, but also indirectly affects sleep through the family’s physical activity behaviors; meanwhile, the significance of the two F-statistics suggests that the father’s physical activity habits is a strong instrumental variable that not only effectively addresses the endogeneity of physical activity and ensures the accuracy of our estimation of the indirect effect. The use of IV-based mediated effects models not only strengthened the causal inference between physical activity and sleep, but also revealed deeper implications for the mechanisms of family physical activity behavior. Please refer to Table 3.

### Stability testing

To further enhance the credibility of the conclusion that physical activity improves adolescent sleep, this paper will conduct a stability test by both replacing the explanatory variables and addressing endogeneity.

### Replacement of implicit variable

Sleep is a multidimensional concept that includes five dimensions of sleep duration, sleep continuity/efficiency, sleep time, wakefulness alertness/drowsiness, and sleep satisfaction/quality, which are appropriate indicators of sleep health (28). Therefore, it is reasonable for this paper to conduct stability tests by replacing the explanatory variable sleep duration for sleep quality, and the researcher can also verify the effect of physical activity on sleep from different perspectives by replacing the explanatory variable. The results showed that after replacing the explanatory variables, physical activity still promoted sleep significantly (*β = −*0.020, *p<0.01*), which suggests that the conclusion that physical activity improves sleep in adolescents has some reliability and robustness. Please refer to Table 4.

**Table 4:** Stability testing table.

Variant	Physical activity	Gender	Birth year	Grade	Total effect
Sleep quality	−0.0203^***^	−0.0195^*^	−0.0493^***^	0.0153	101.0281^***^
	(−0.003)	(−0.012)	(−0.005)	(−0.018)	(−9.365)
*N*	21,147				
*R2*	0.069				

*, ** and *** indicate significance at the 10, 5and 1% statistical levels, respectively; robust standard errors are in parentheses.

### Addressing endogenous issues

There may be a bidirectional causal relationship between physical activity and sleep, i.e., physical activity can have a facilitating effect on sleep, and sleep can influence physical activity. Therefore, this paper controls the bidirectional causal relationship between physical activity and sleep by instrumental variable method and the results are shown in the table. Model 2 and Model 3 in Table 2 are the instrumental variable test and the instrumental variable test after adding the control variables, respectively. As can be seen from Model 2, the instrumental variable, father physical exercise habit, has a significant contribution to sleep, which indicates a strong correlation between the two, but the instrumental variable may also have the problem of endogeneity, therefore, this paper also examines the weak instrumental variable test, the test of exogeneity, and the Hausman test through the endogeneity problem of instrumental variables. The results show that the coefficient value of the weak instrumental variable is 0.2080 (*β =* 0.208, *p<0.01*), which indicates that the instrumental variables selected in this paper are relatively strong and provide valid estimates. Also, the effect of physical activity on sleep was consistent in the test of exogeneity (*β =* 0.208, *p<0.01*), which also suggests that the physical activity variable is statistically exogenous and that the estimates are plausible. The *p*-value for physical activity was significant in the Hausman test (*β =* 0.052, *p<0.01*), which suggests that a fixed-effects model is more appropriate for this study than a random-effects model because there are a number of fixed, unobserved individual characteristics that influence sleep. An in-depth analysis of the weak instrumental variable test, exogeneity test and Hausman test leads us to conclude that the model and methodology used in this study are statistically robust. Please refer to Table 5.

**Table 5:** Instrumental variables regression model test table.

Inspect	Weak instrumental variables test	Exogeneity test	Hausmann test	
Physical exercise	0.208^***^ (−9.57)	0.208^***^ (−9.57)	0.052^***^ (−0.004)	0.208^***^ (−0.022)
Aggregate effect	6.893^***^ (−96.35)	6.893^***^ (−96.35)	7.402^***^ (−0.017)	6.893^***^ (−0.072)
*N*	20,956			

*, ** and *** indicate significance at the 10, 5and 1% statistical levels, respectively; robust standard errors are in parentheses.

### Heterogeneity analysis

As adolescents enter adolescence, there is much concern about uneven and inadequate development among their individuals, which makes it likely that there are significant differences in the effects of physical activity on sleep in boys and girls (29). Although different groups have different purposes and motives for participating in physical exercise, the functions of physical exercise, such as recreation, strengthening the body, relieving fatigue, and preventing diseases, are important purposes for all groups to participate in physical exercise, and with the fulfillment of these purposes, physical exercise will improve the quality of sleep both psychologically and physiologically. This paper further investigates the changing relationship between physical activity and sleep in boys and girls and whether there is a significant difference in the effect of physical activity on sleep in boys and girls. The results showed that the average sleep duration of men was greater than that of women at the same level of exercise, which may be due to the differences in sleep requirements between men and women and that men may need more recovery time to repair and rebuild their muscle tissues, especially after high-intensity physical activity. The results showed that physical activity had a significant effect on sleep in all groups and that girls’ participation in physical activity had a greater effect on sleep than boys’ due to differences in girls’ and boys’ strategies for coping with stress. Please refer to Table 5.

## Discussion

The relationship between physical activity and sleep quality in adolescents has received extensive attention in existing research. According to the results of the study, physical activity had a significant positive effect on the quality of sleep-in adolescents, especially in terms of increased sleep duration. The coefficient of influence of physical activity on adolescents’ sleep analyzed by Model 1 is 0.052, implying that for every unit increase in adolescents’ physical activity participation, the number of hours of sleep will increase by 5.2%. This finding is consistent with numerous existing studies showing that physical activity is effective in improving sleep problems in adolescents. For specific analysis, physical exercise (32), a comfortable sleep environment (33), regular work and rest schedules (34), mindfulness (35), nap time (36), sleeping pills (37), and medical treatment can improve sleep in adolescents, but the study found that exercise has a more significant effect on sleep improvement, and may be a more appropriate long-term intervention compared to hypnotic drugs (38). Physiologically, exercise can influence sleep in adolescents by improving cardiorespiratory fitness, body temperature, circadian rhythms, cytokine concentrations, and brain neuropeptides (30, 31). Moreover, regular physical activity can help regulate the body’s biological clock, improving sleep quality, reducing the time it takes to fall asleep, and decreasing the frequency of waking up during the night (32). Psychologically, physical activity also reduces stress and anxiety in adolescents, thus improving their sleep. Self-regulation theory suggests that adolescents need to continually improve their willpower and emotional regulation during the process of social adaptation, and physical activity provides an opportunity for the development and improvement of their psychological regulation skills (9). Therefore, moderate physical exercise is believed to help adolescents alleviate psychological discomfort and sleep disorders (39) and to have a significant effect on the development of a positive adolescent psyche (Table 6).

**Table 6:** Heterogeneity analysis table.

Variant	Boys	Girls
	Sleep	
Physical activity	0.041^***^ (−0.006)	0.057^***^ (−0.006)
Total effect	7.523^***^ (−0.025)	7.299^***^ (−0.022)
*N*	10,661	10,295
*R2*	0.004	0.009

*, ** and *** indicate significance at the 10, 5and 1% statistical levels, respectively; robust standard errors are in parentheses.

This study proposes that fathers’ physical activity habits affect their children’s physical activity attitudes and behaviors, but have no direct effect on their children’s sleep quality, and that this effect can only be achieved through their children’s physical activity habits, which is consistent with the results of the weak instrumental variable test, the exogeneity test, and the Hausman test in Table 5. The addition of the Model 2 instrumental variable (father’s physical activity habits) increased the coefficient of the effect of physical activity on sleep from 0.052 to 0.208, further demonstrating the validity of the instrumental variable. This is due to the fact that fathers’ involvement in physical activity sets a role model for adolescents, helping them to establish healthy physical activity habits37 and indirectly affecting their sleep (40). Research has shown that fathers’ involvement in adolescent physical activity is closely related to adolescents’ exercise habits, and that this mechanism of father-child interaction has a positive impact on the shaping of adolescents’ health behaviors (41). This is similar to the “role model effect” in psychology, in which parents, especially fathers, are often seen as role models and role models for their children, as their regular and healthy lifestyles in daily life are positively observed and imitated by their children. Although fathers’ physical activity habits served as a valid instrumental variable in this study, future research could further explore other potential instrumental variables to increase the robustness and credibility of the findings. For example, studies have shown that mothers’ participation in physical activity has a different impact on adolescents than fathers’, especially in family health decision-making, where mothers tend to play a more active role. Therefore, in the future, mothers’ physical activity behaviors can be used as an instrumental variable in comparative analyses to explore the effects of mothers’ physical activity behaviors on adolescents’ exercise habits and sleep quality. In addition to considering parental physical activity habits in isolation, overall family health behaviors (e.g., family sports climate, family sports environment, family sports perceptions, etc.) can also be studied as a potential instrumental variable.

This study also found that the mediating effect of physical activity on sleep accounted for as much as 84.62% of the total effect, while the direct effect accounted for only 15.53% of the total effect. This result suggests that adolescent physical activity indirectly improves adolescent sleep quality by influencing family physical activity behaviors. This finding is consistent with social learning theory, in which parents of adolescents learn new behavioral patterns by observing and imitating their adolescents’ behaviors (42). When adolescents are physically active or show their parents the results of their physical activity, it motivates their parents to participate in physical activity with their children, thus improving the health habits of the entire family (42). This mediating pathway reveals the dynamic transmission and interaction mechanisms of health behaviors within the family. In addition to this, follow-up analyses showed that family sports behaviors affect adolescents’ sleep. As the primary environment in which adolescents grow up, the lifestyle of the family is extremely important in influencing adolescents (43). First, those families that value and regularly participate in physical activity not only shape a healthy outlook on life for their youth, but also foster a regular physical activity habit for them. This habit directly affects adolescents’ perceptions of sleep, which in turn positively influences their sleep quality (34). Second, adolescents who grow up in an environment that emphasizes the importance of physical activity and healthy living concepts tend to take positive steps to cope when faced with sleep disorders (44). Such proactive measures may be initiating physical activity to improve sleep quality or communicating with parents for solutions. In summary, such adolescents are more likely to cope with sleep problems by making lifestyle adjustments and seeking family support than other adolescents. Finally, our study also confirms that adolescents’ physical activity behaviors can indirectly affect their own sleep by influencing their family’s physical activity behaviors and, ultimately, their own sleep. The family as a whole plays a particularly important role in promoting healthy adolescent development (45). Psychodynamic theory suggests that a physically active adolescent may be a catalyst for health behavior change in other members of the family, thereby influencing the physical activity habits of the entire family (42). This changed family physical activity habit creates a more positive home environment climate that promotes emotional connection and support among family members, which has an indirect positive effect on adolescent sleep quality. This indirect effect is further explained by the Stages of Change Theory (46), in which adolescents’ self-management, perseverance, and health improvement through physical activity (47) can be powerful examples to motivate family members to change their own behaviors, thus shifting family members’ awareness of participating in physical activity from the stage of contemplation to the stage of action (42). Ultimately, these changes not only improve the quality of sleep for teens, but also improve sleep hygiene practices for the entire family, such as regular work schedules and reduced evening screen time.

## Limitations

On the basis of existing theoretical and empirical studies, this paper tries to analyze the effect of physical exercise on sleep in adolescents, which is of some value to the study of adolescent sleep health, but there are some limitations in this paper due to the limitation of data and methods. For one thing, the explanatory variable sleep includes only sleep duration, but sleep is a multidimensional concept that includes five dimensions: sleep duration, sleep continuity/efficiency, sleep time, wakefulness alertness/drowsiness, and sleep satisfaction/quality. Therefore, data selectivity bias poses a challenge to the empirical part of this paper and prevents a more in-depth and comprehensive discussion of the relationship between physical activity and sleep in adolescents. Second, the factors influencing physical activity on sleep are complex, including the effects of health status, stress, family culture, and environment, but the analysis in this paper is still deficient at the level of comprehensive explanation. It is expected that future research will be further explored based on the multidimensionality of the data and the comprehensiveness of the analysis, and it is suggested that more valuable findings will be obtained.

## Conclusion

There is a significant correlation between physical activity and sleep. The instrumental variable father physical activity habits made the positive effects of physical activity on sleep more pronounced after controlling for potential endogenous biases. Family physical behavior has a significant mediating role in the influence of physical activity on adolescent sleep, and this indirect mediating effect further enhances the improvement of sleep by physical activity.

### Suggestion

Based on this, the paper also makes the following recommendations:

- To increase opportunities for physical exercise for young people: the State should reform school education policies to increase the number of hours of physical education classes, so that students can master 1–2 sports skills in physical education classes to meet the needs of different students for physical exercise; the community should provide young people with more opportunities for physical exercise, including community sports clubs and community games.
- Publicity and education on sleep should be strengthened: through school education and social publicity, parents and adolescents should be made aware of the importance of sleep, especially the role of physical exercise in improving sleep quality, and families should be encouraged to adopt an active and healthy lifestyle.
- Family sports days and a family sports culture should be actively created: parents should set an example for young people by actively participating in sports, encouraging the whole family to participate in sports activities together, and arranging regular days of common family sports, such as family hiking, bicycling and mountain climbing; Parents should establish a positive exercise culture, such as by setting exercise goals together, participating in outdoor adventures, or establishing bedtimes at home.
- To pay attention to the emotional needs of adolescents: Parents should create a living environment conducive to the growth of adolescents, actively listen to their thoughts and feelings, provide them with emotional support and guidance, and help them to effectively manage the pressure of learning and life.
- Individual adolescents should establish a healthy concept of life: adolescents should choose appropriate sports activities according to their personal interests, such as swimming, soccer, basketball, etc., and incorporate physical exercise into their daily lives, while engaging in at least moderate-intensity physical exercise every week; Teenagers should reasonably arrange study and rest time, avoid staying up late, and ensure sufficient sleep time every night to promote the recovery of the body and brain; teenagers should take the initiative to learn about sleep hygiene and correct bad sleep hygiene habits.
- Regular monitoring and feedback: Through home-school cooperation, the quality of sleep and physical activity of adolescents is regularly monitored, and feedback and advice are provided to help them establish and maintain healthy living habits.

## Data Availability

The original contributions presented in the study are included in the article/supplementary material, further inquiries can be directed to the corresponding author.
